# Snake venom natriuretic peptides: Potential molecular probes

**DOI:** 10.1186/2050-6511-16-S1-A87

**Published:** 2015-09-02

**Authors:** Sindhuja Sridharan, R Manjunatha Kini

**Affiliations:** 1 Department of Biological Sciences, National University of Singapore, Singapore-117543, Singapore; 2 Department of Biochemistry and Molecular Biophysics, Virginia Commonwealth University, Richmond, VA 23298, USA; 3 School of Pharmacy and Medical Sciences, University of South Australia, Adelaide, SA 5000, Australia

**Keywords:** Natriuretic peptides, Snake venom, cGMP, vasodilation, nitric oxide

## 

Natriuretic peptides (NPs) play a vital role in the regulation of pressure-volume homeostasis. They mediate vasodilation and increase water-electrolyte excretion through kidneys by activating membrane bound guanylyl cyclase receptors (NPR). Apart from the three mammalian isoforms, snake venom NPs with distinct pharmacological functions have been characterized. This study describes the molecular mechanism and physiological action of a novel NP from krait (*Bungarus flaviceps*) venom called KNP. This peptide has the conserved 17-residue NP ring with a 38-residue long C-terminal tail which has propensity to form an α-helix. Intravenous infusion of KNP in rats resulted in sustained and prolonged reduction in blood pressure without renal effects compared to the mammalian counterpart. It mediates vasodilatory function via endothelium in a NPR-independent manner. Deletion mutant studies show the presence of two active segments in KNP, namely; K-Ring and K-Helix. K-Ring functions similar to a classical NP; through its interaction with NPR, thereby evoking an endothelium-independent vasorelaxation response. Interestingly, K-Helix induces vasorelaxation through endothelium via NO-dependent pathways. Thus, despite the presence of a functional NP ring, the putative helical segment of KNP seems to drive the function of this NP. Further, infusion of K-Ring in rats shows similar vascular effects as mammalian NPs without diuresis. Despite the ability to activate NPR, K-Ring showed tissue-specific activity. Substitution of two amino acid residues in K-Ring restored renal function, suggesting the importance of conformational plasticity of NPs in activating downstream response. Thus, KNP provides a new paradigm in the understanding of NP biology and may open avenues for design and development of novel NP-based therapeutics.

